# Cross-Sectional Associations of Neighborhood Perception, Physical Activity, and Sedentary Time in Community-Dwelling, Socioeconomically Diverse Adults

**DOI:** 10.3389/fpubh.2019.00256

**Published:** 2019-09-13

**Authors:** Sophie E. Claudel, Eric J. Shiroma, Tamara B. Harris, Nicolle A. Mode, Chaarushi Ahuja, Alan B. Zonderman, Michele K. Evans, Tiffany M. Powell-Wiley

**Affiliations:** ^1^Social Determinants of Obesity and Cardiovascular Risk Laboratory, National Heart, Blood, Lung Institute, National Institutes of Health, Bethesda, MD, United States; ^2^Laboratory of Epidemiology and Population Science, National Institute on Aging, National Institutes of Health, Bethesda, MD, United States

**Keywords:** physical activity, sedentary time, racial/ethnic disparities, neighborhood, perceived crime, HANDLS

## Abstract

**Background:** Little is known about the role of perceived neighborhood environment as a determinant of physical activity (PA) and sedentary time (ST) in understanding obesity-related health behaviors. We focus on a biracial, socioeconomically diverse population using objectively measured ST, which is under-represented in the literature.

**Methods:** We examined the association between self-reported neighborhood perception (Likert-scale questions), PA using the Baecke questionnaire, and both non-sedentary time and ST using accelerometry from wave 4 of the Healthy Aging in Neighborhoods of Diversity across the Life Span (HANDLS) study (*n* = 2,167). After applying exclusion criteria, the sample size was *n* = 1,359 for analyses of self-reported PA and *n* = 404 for analyses of accelerometry data. Factor analysis identified key neighborhood characteristics to develop a total neighborhood perception score (NPS). Higher NPS indicated less favorable neighborhood perception. Linear regression was used to determine the relationship between NPS, PA, non-sedentary time, and ST.

**Results:** Complete data were available for *n* = 1,359 [age 56.6(9.0) years, 59.5% female, 62.2% African American] for whom we identified four neighborhood perception factors: (1) concern about crime, (2) physical environment, (3) location of violent crime, and (4) social environment. Worsening perception of the overall neighborhood [β = −0.13 (SE = 0.03); *p* = 0.001], the physical environment [−0.11 (0.05); *p* = 0.03], and the social environment [−0.46 (0.07); *p* < 0.0001] were associated with decreased PA. Worsening perception of the overall neighborhood [1.14 (0.49); *p* = 0.02] and neighborhood social environment [3.59 (1.18); *p* = 0.003] were associated with increased ST over the day. There were no interactions for race, sex, poverty status, or economic index.

**Conclusion:** Poor overall neighborhood perception, perceived social environment, and perceived neighborhood physical environment are associated with PA and ST in a multi-racial, socioeconomically diverse cohort of urban adults.

**Clinical Trial Registration:** The HANDLS study is registered at ClinicalTrials.gov as NCT01323322.

## Introduction

In the United States, 25% of deaths are attributable to cardiovascular disease, making it the leading cause of death for both men and women (1). Nearly 50% of Americans have a significant risk factor for cardiovascular events, including hypertension, hyperlipidemia, and smoking; yet few are actively addressing these risks with health behavior change (1). Physical activity (PA) has been repeatedly shown to reduce risk of cardiovascular events, decrease overall mortality, and ameliorate health, leading multiple expert panels to recommend increased PA and decreased sedentary time (ST) as important preventive measures (2). Sedentary time is critical for intervention as it is an independent risk factor for cardiometabolic disease (3, 4) and, with accumulating evidence, all-cause mortality (5, 6). However, individual behavior cannot be considered independently of its environmental context, which may be exerting formidable influence on decision-making through perception and opportunity.

The neighborhood environment is undoubtedly a key determinant of cardiovascular health, as it has been repeatedly associated with increasing body mass index (7), cardiovascular events (8), and incident diabetes (9, 10). Neighborhood factors that influence health include crime (6, 7), perceived safety (11), physical attributes (graffiti, lighting, structural damage, etc.) (12), social cohesion (13), and walkability (10). A recent review highlights the constructs underlying neighborhoods and cardiometabolic health (14). Social cohesion refers to the collective efficacy that underlies building shared community values to advance toward common goals (15–17). Neighborhood disorder refers to physical decay and fear that negatively impact the neighborhood environment (15, 18). Walkability is the ability of the physical environment to facilitate outdoor recreational activities, such as active transport (10, 19). The more notable debate regarding these concepts is whether research should emphasize objective or perceived measures of neighborhood condition. Several studies demonstrate resident perception of these factors as more predictive of health outcomes than objective measures (7, 11, 20, 21).

Although many studies have focused on neighborhood barriers to PA, few have looked at factors that promote sedentary behavior (22). Those that have evaluated the relationship between neighborhood factors and ST have reported mixed results, likely due to variable neighborhood measures and inconsistent operational definitions of ST. Use of objective measures of ST is even more rare. Kozo and colleagues investigated accelerometry and self-reported ST to evaluate the relationship between ST and neighborhood walkability, but did not consider other neighborhood characteristics (23). Similarly, Van Dyck and colleagues examined transit-related ST in relation to neighborhood walkability and transportation resources, but did not measure other neighborhood conditions and relied entirely on self-reported ST (24). Despite the discrepancies in measurement and analysis, preliminary findings support the role of neighborhood environment on ST (25, 26) and health outcomes such as blood pressure, resting heart rate, and body mass index (BMI) (21, 25). Further studies are needed to evaluate the role of the neighborhood environment on health behaviors within diverse populations, using objective measures of ST, and considering multiple neighborhood characteristics.

Using data from the Healthy Aging in Neighborhoods of Diversity across the Life Span (HANDLS) study of African American and white, socioeconomically diverse, community-dwelling adults, we explore the neighborhood environment as a key precipitant of CVD risk through its ability to decrease PA and promote ST. This study aims to investigate the cross-sectional relationship between perception of neighborhood environment, PA, and ST, as well as the potential moderating effects of race, sex, and socioeconomic status. We expect that those who perceive a worse neighborhood environment will be less likely to engage in PA and more likely to engage in sedentary behavior.

## Methods

### HANDLS Study

The HANDLS study is a cohort (*n* = 3,720) of white and African American socioeconomically diverse individuals in Baltimore City, Maryland 30–64 years old at baseline. The participants are a fixed cohort recruited from 13 neighborhoods selected by area probability sampling. As a longitudinal study, HANDLS collected consecutive waves of data every 4–5 years with interim analyses. Waves 1 through 4 were completed between August 2004 and September 2017, with Wave 4—used here—occurring from September 2013 to September 2017. The study design and methodology have been previously described in detail (27). The National Institute of Environmental Health Sciences institutional review board approved the study and all participants signed written, informed consent.

Secondary data from the HANDLS study were analyzed for this project. Using HANDLS Wave 4 data (*n* = 2,167), we excluded participants who refused to answer the entire neighborhood questionnaire (*n* = 591) or entire PA questionnaire (*n* = 103), as well as those who lacked complete covariate data (*n* = 208). This resulted in a sample of 1,359 for analysis of PA. A subset of Wave 4 participants were offered the opportunity to participate in the accelerometry. The sample size is limited in these analyses by both the smaller cohort who participated and by missing covariate data. The final analytic sample for the accelerometry cohort was *n* = 404. HANDLS Wave 4 was the only wave with accompanying accelerometer data, therefore, the analyses were conducted cross-sectionally rather than longitudinally.

### Neighborhood Questionnaire

The exposure of interest, neighborhood perception score (NPS), was derived via principal components factor analysis from a neighborhood questionnaire completed by HANDLS participants. Response options were scaled on a 5-point Likert scale. The questionnaire consisted of two questions on neighborhood accessibility, five questions on neighborhood social cohesion, three questions on neighborhood social conscience, 11 questions on neighborhood disorder, and 29 questions on neighborhood crime. Neighborhood social cohesion, social conscience, and disorder were assessed using dimensions suggested by Sampson et al. (15, 28). The remaining neighborhood violence questions were developed by a HANDLS collaborator and expert in the field.

### Measures of Physical Activity and Sedentary Time

Self-reported Likert scale responses to the Baecke Physical Activity Questionnaire were reverse coded as needed and summed into individual category scores (Work, Sport, and Leisure) and then summed again for a total PA score. Work, Sport, and Leisure PA are standard indices of the Baecke Questionnaire and are created by summing responses to the questions included in each section of the questionnaire, as described by Baecke et al. (29). Leisure time PA (LTPA) was defined as the sum of Sport and Leisure PA. A higher combined score indicates a greater level of PA. This questionnaire has been repeatedly validated in numerous populations (30–32). However, there is no standard conversion to minutes per week of PA.

ST and non-sedentary time were measured by an ActiGraph GT3X+ accelerometer in a subset of the Wave 4 participants (*n* = 760). The wrist location of the ActiGraph has been shown to have increased participant adherence for 24-h wear compared to both hip-based locations and the adhesively attached leg of the ActivPAL. Additionally, ActiGraph has a longer battery life. Participants were asked to wear a wrist-worn ActiGraph 24 h per day (except when bathing, showering, or swimming) for 1 week. Accelerometer data were aggregated into 60-s epochs and then screened for non-wear using a standard algorithm to detect sustained periods of non-movement (33). Accelerometer data were considered to be valid if participants had more than 10 h per day of wear on at least 4 days. Accelerometer-assessed sedentary time was defined as the number of minutes when the accelerometer registered <1,853 vector magnitude counts (34). A vector magnitude count is an aggregated measure of acceleration across all three axes. Non-sedentary time was used as a proxy measure for objective PA in these participants. Accelerometer data were further categorized by time of day as total waking hours (5 a.m.−11 p.m.); exploratory analyses were conducted with three additional categories: morning hours (5 a.m.−9 a.m.), working hours (9 a.m.−5 p.m.), and evening hours (5 p.m.−11 p.m.).

### Covariates

The covariates used in the analyses were collected during the HANDLS study visit via interview and questionnaire. The categorical covariates include sex (women vs. men), race/ethnicity (African American vs. Non-Hispanic White), poverty status (above vs. below 125% of the federal poverty level), education [less than high school vs. high school/General Educational Development (GED) and above], and length of residence in the neighborhood (<1 year vs. ≥1 year). Continuous covariates include age (years), BMI (kg/m^2^), and neighborhood economic index based on 2012–2016 American Community Survey data [NEI; see Mode et al. (35)]. Neighborhood economic index was used to provide a measure of objective neighborhood environment in the analysis. Covariates were selected following review of the literature (35–37).

### Statistical Analysis

The neighborhood perception score (NPS) was calculated from the neighborhood questionnaire using the following analytic technique. Principal axis factoring was used to identify common themes (factors) from the neighborhood questionnaire. The sample size was assessed to be sufficient for factor analysis based on prior research (38). Promax (oblique) rotation was applied. A loading score of 0.40 was required for inclusion in the factor and the minimum eigenvalue was set at 1. Neighborhood perception scores were computed using only the items that loaded into factors. The numerical value of each Likert-scale response was summed to create a total neighborhood perception score (NPS) and factor-specific NPS. Cronbach's alpha measured the internal consistency of each factor; only those with a value above 0.70 were considered acceptable. A higher NPS represents a worse perception of the neighborhood environment. Analysis of Kurtosis and Skew demonstrated that NPS was normally distributed.

Descriptive statistics of participant characteristics were calculated and evaluated for correlation to total NPS using pairwise correlation coefficients. Since the data are a multilevel structure (i.e., individuals nested within census tracts), an intraclass correlation coefficient (ICC) was calculated for an intercept only model. The ICC was 2.7%, indicating a low proportion of the variability lies within census tracts. Although ICC is not the only available indicator for determining the need for multilevel modeling, it informed the decision to pursue simple regression models instead of multilevel modeling (39–41). Multivariable linear regression modeling evaluated the influence of NPS on PA, LTPA, non-sedentary time, and ST. Models were adjusted for age, BMI, race (referent = white), poverty status (referent = above poverty level), education (referent = below high school/GED), NEI, and length of residence at the same address (referent ≤ 1 year). Multicollinearity of the variables was examined using tolerance and variance inflation factors (VIF), and independent variables (i.e., age, race, sex, BMI, income, education, length of residence, poverty status, NEI) were not found to be collinear (VIF <2.0). After adjustment for covariates, the sample size for the PA and LTPA was *n* = 1,359 and the sample for ST and non-sedentary time was *n* = 404, due to missing covariate data. Interaction terms between NPS and race, sex, NEI, and poverty status were evaluated. All analyses were conducted in Stata/IC Version 12.1 (StataCorp, College Station, TX).

## Results

Participant socio-demographic, health, and neighborhood characteristics are shown in Table 1. Total NPS ranged from 42 to 140, with a median value of 87. The sample was ~60% female, 62% African American, and had a mean age of 56.1 years (SD 9.0) and mean BMI of 31.0 kg/m^2^ (SD 7.7). None of the socio-demographic or activity variables were strongly correlated to total NPS, as all correlation coefficients were <0.18 (Table 1). Correlations between the factor scores were also assessed and found to be minimal (data not shown).

**Table 1:** Correlations between participant characteristics and total neighborhood perception score for the analytic cohort (*n* = 1,359).

	**Estimate**	**Correlation with NPS**	***p-*value**
NPS score range	42–140		
**SOCIODEMOGRAPHIC VARIABLES**			
Age (Years)	56.1 (9.0)	−0.12	<0.0001
Sex		0.07	0.01
Women	809 (59.5)		
Men	550 (40.5)		
Race		−0.18	<0.0001
African American	845 (62.2)		
White	514 (37.8)		
Poverty Status		0.06	0.03
Below Poverty Level	492 (36.2)		
Above Poverty Level	867 (63.8)		
Education		−0.02	0.48
12th grade and below	402 (29.6)		
High School/GED and above	957 (70.4)		
BMI (kg/m^2^)	31.0 (7.7)	−0.003	0.91
Length of Residence		−0.01	0.74
<1 year	161 (11.9)		
≥1 year	1,198 (88.2)		
Neighborhood Economic Index (NEI)	−3.6 (4.3)	−0.13	<0.0001
**ACTIVITY VARIABLES**			
Physical Activity (Baecke)			
Work PA Score^a^	13.9 (10.6)	−0.05	0.06
Leisure Time PA Score^b^	15.2 (10.3)	−0.08	0.003
Total PA Score^c^	28.4 (16.1)	−0.09	0.001
Average Sedentary Time (min)			
Total Waking Hours (5 a.m.−11 p.m.)	602.8 (136.5)	0.14	0.006
Morning Hours (5 a.m.−9 a.m.)	162.4 (50.9)	0.13	0.008
Working Hours (9 a.m.−5 p.m.)	242.0 (67.8)	0.12	0.01
Evening Hours (5 p.m.−11 p.m.)	204.4 (52.8)	0.06	0.20
Percent Time Spent Sedentary			
Total Waking Hours (5 a.m.−11 p.m.)	59.4 (12.1)	0.13	0.007
Morning Hours (5 a.m.−9 a.m.)	73.1 (17.8)	0.15	0.003
Working Hours (9 a.m.−5 p.m.)	52.3 (14.7)	0.11	0.02
Evening Hours (5 p.m.−11 p.m.)	60.4 (13.8)	0.07	0.17

*Estimate represents Mean (SD) or N (%), as appropriate*.

a *Total possible work PA score = 38*.

b *Total possible leisure time PA score = 50*.

c *Total possible overall PA score = 88*.

The results of the principal component factor analysis are shown in Table 2. The analysis yielded four factors that together explained 93.7% of the variance. The four factors were interpreted as: (1) concern about specific types of crime, (2) physical environment, (3) location of violent crime, and (4) social environment, with Cronbach's alpha coefficients of 0.96, 0.93, 0.87, and 0.83, respectively. As shown in Table 2, 13 questions defined worrying about specific types of crime, 11 questions defined perceptions of neighborhood physical environment, five questions defined perceptions of where violent crime occurs, and seven questions defined perceptions of neighborhood social environment.

**Table 2:** Rotated factor loading scores and mean Likert scale response to the questions that loaded into each factor (*n* = 1,359).

**Factor**	**Question pertaining to:**	**Likert scale response mean (SD)**	**Factor 1**	**Factor 2**	**Factor 3**	**Factor 4**
Concern about specific types of crime (Factor 1)	Violent crime inside neighbors' homes	2.68	**0.54**	−0.11	0.21	0.02
	Murders near where you live	2.21	**0.82**	0.03	0.05	−0.02
	Shootings near where you live	2.13	**0.83**	−0.02	0.03	0.02
	Rapes or sexual assaults near where you live	2.24	**0.94**	0.03	−0.09	0.03
	Robberies near where you live	2.13	**0.86**	0.03	0.03	−0.009
	Car-jackings near where you live	2.44	**0.93**	0.02	−0.10	0.08
	Aggravated assaults (serious harm) near where you live	2.31	**0.96**	0.005	−0.04	0.03
	Common assaults (minor harm) near where you live	2.32	**0.92**	0.02	0.009	0.02
	Residential burglaries near where you live	2.21	**0.85**	0.04	0.03	0.001
	Violent crime inside neighbors' homes	2.48	**0.93**	0.008	−0.11	0.02
	Violent crime on your street	2.44	**0.80**	−0.05	0.04	−0.02
	You being victimized (mugged, robbed, assaulted)	2.55	**0.62**	0.003	0.16	−0.13
	Your home being burglarized	2.58	**0.46**	−0.01	0.20	−0.14
Physical environment (Factor 2)	Graffiti in neighborhood	2.32	0.03	**0.65**	−0.05	0.07
	Litter in neighborhood	3.00	0.04	**0.64**	−0.03	0.07
	Drug dealers, drug users, or drunks in neighborhood	2.73	−0.06	**0.78**	0.06	−0.007
	Unemployed adults loitering in neighborhood	2.62	−0.003	**0.82**	0.02	0.008
	Gang activity in neighborhood	1.92	−0.03	**0.73**	0.02	0.02
	Disorderly teens or children in neighborhood	2.43	−0.001	**0.76**	0.03	0.05
	Prostitution in neighborhood	1.87	0.003	**0.64**	0.04	0.06
	Vacant or abandoned buildings in neighborhood	2.57	−0.008	**0.85**	0.04	−0.08
	Broken windows in neighborhood	2.23	−0.02	**0.87**	0.09	−0.07
	Serious crimes (assault, mugging, robbery)	2.37	−0.005	**0.68**	−0.09	0.01
	Houses or yards not kept up in neighborhood	2.65	0.02	**0.72**	0.02	0.009
Location of violent crime (Factor 3)	Violent crime on your street	2.61	0.26	−0.22	**0.45**	−0.05
	Violent crime on adjacent streets	2.59	0.31	−0.21	**0.47**	−0.03
	Violent crime several streets away	2.46	0.20	−0.13	**0.66**	−0.007
	Violent crime in other neighborhoods	2.12	0.06	0.09	**0.83**	0.05
	Violent crime across the city as a whole	1.93	0.07	0.13	**0.76**	0.09
Social environment (Factor 4)	Neighbors do not get along	2.50	−0.08	0.14	0.09	**0.44**
	Neighbors are not willing to help each other	2.39	−0.06	−0.11	0.04	**0.72**
	Not a close knit neighborhood	2.74	0.03	−0.07	−0.05	**0.70**
	Neighbors cannot be trusted	2.90	−0.10	0.06	0.07	**0.62**
	Neighbors do not take action if children spray-paint	2.37	0.02	0.22	0.05	**0.53**
	Neighbors do not take action if children are disrespectful	2.51	0.08	0.04	−0.01	**0.70**
	Neighbors do not take action if there is a fight	2.48	0.03	0.06	0.04	**0.66**
		Eigenvalue	14.02	5.19	1.83	1.33
		Common variance explained	58.7%	21.7%	7.7%	5.6%
		Cronbach's α coefficient	0.96	0.93	0.87	0.83

*Bold values indicate loading scores ≥0.4, which qualified the item for inclusion in the factor*.

**Figure 1:**
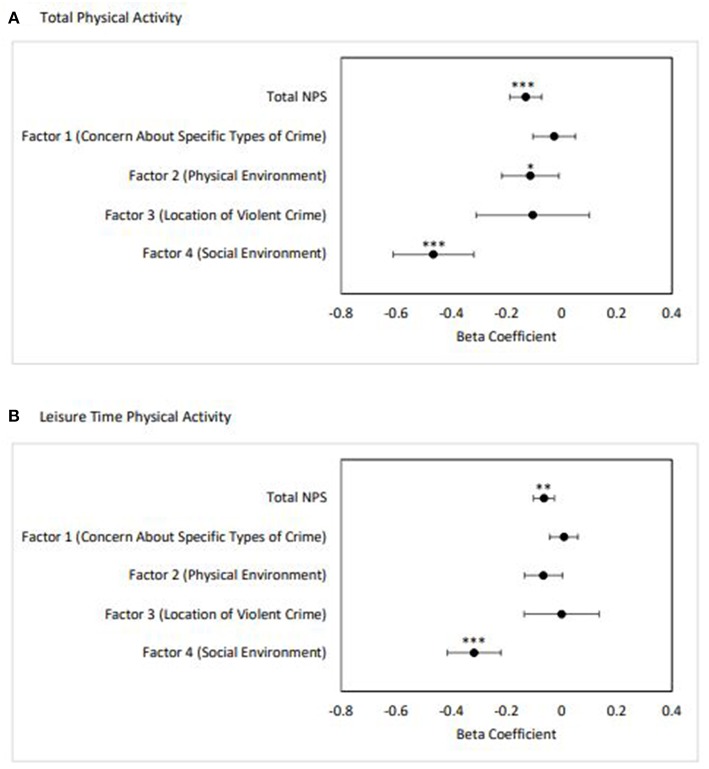
Adjusted linear regression results of total and factor-specific NPS on self-reported physical activity (*n* = 1,359). **(A)** Total PA, **(B)** Leisure time PA. Error bars represent 95% confidence interval. Adjusted for age, BMI, sex, race, poverty status, education, NEI, and length of residence in the neighborhood (^*^p < 0.05, ^**^p ≤ 0.001, ^***^p < 0.0001).

Figure 1 shows the beta coefficients (β) and 95% confidence intervals of the regression results for total and factor-specific NPS on self-reported PA. For total PA, worsening overall neighborhood perception (β = −0.13; SE = 0.03; *p* = 0.001), perception of the neighborhood physical environment (Factor 2; β = −0.11; SE = 0.05; *p* = 0.03), and perception of the neighborhood social environment (Factor 4; β = −0.46; SE = 0.07; *p* < 0.0001) were associated with lower levels of PA. For leisure time PA, worsening overall neighborhood perception (β = −0.06; SE = 0.02; *p* = 0.001) and perception of the neighborhood social environment (Factor 4; β = −0.32; SE = 0.05; *p* < 0.0001) were associated with decreased LTPA. There were no significant interactions for sex, race, or socioeconomic status. The regression coefficients for all covariates in each model for PA and LTPA are included in Supplemental Tables 1, 2, respectively.

Table 3 shows the regression results of total and factor-specific NPS on objectively measured ST. Worsening perception of the overall neighborhood environment was associated with increased ST over the entire waking day (β 1.14; SE 0.49; *p* = 0.02). Worsening perception of the neighborhood social environment (Factor 4) was associated with increased ST over the entire day (β 3.59; SE 1.18; *p* = 0.003). There were no significant interactions for sex, race, or socioeconomic status.

**Table 3:** Adjusted linear regression results of total and factor-specific NPS on accelerometer-measured sedentary time for the entire waking day (*n* = 404).

	**Model 1**		**Model 2**		**Model 3**		**Model 4**		**Model 5**	
	**β**	**SE**	**β**	**SE**	**β**	**SE**	**β**	**SE**	**β**	**SE**
Total NPS	**1.14**	0.49	–	–	–	–	–	–	–	–
Factor 1 Score	–	–	0.03	0.96	–	–	–	–	–	–
Factor 2 Score	–	–	–	–	0.86	0.85	–	–	–	–
Factor 3 Score	–	–	–	–	–	–	2.48	1.75	–	–
Factor 4 Score	–	–	–	–	–	–	–	–	**3.59**	1.18
Age	**2.83**	0.74	**2.72**	0.74	**2.79**	0.74	**2.71**	0.74	**3.02**	0.74
Sex	**33.40**	13.83	**38.18**	13.80	**37.22**	13.78	**37.58**	13.73	**34.22**	13.67
BMI	**2.94**	0.87	**3.14**	0.88	**3.18**	0.87	**3.11**	0.87	**3.00**	0.87
Race	−12.98	14.49	−14.42	14.74	−10.72	14.98	−17.51	14.71	−8.41	14.54
Poverty Status	15.64	13.84	17.74	13.90	16.91	13.90	16.86	13.88	14.89	13.78
Education	4.09	14.07	5.60	14.21	6.15	14.14	5.61	14.11	6.01	13.99
Residence	−27.65	20.95	−29.82	21.11	−30.86	21.06	−28.08	21.05	−29.12	20.83
NEI	−0.28	1.60	−0.54	1.64	0.03	1.70	−0.86	1.62	0.34	1.61
Intercept	**255.94**	69.56	**355.70**	56.50	**328.29**	61.16	**328.10**	57.96	**279.38**	59.59

*Bold indicates p < 0.05*.

*Model 1: Exposure variable is Total NPS*.

*Model 2: Exposure variable is Factor 1 (Concern about specific types of crime)*.

*Model 3: Exposure variable is Factor 2 (Physical environment)*.

*Model 4: Exposure variable is Factor 3 (Location of violent crime)*.

*Model 5: Exposure variable is Factor 4 (Social environment)*.

Further exploratory analyses were conducted to evaluate ST during discrete periods of the day (morning hours, working hours, and evening hours). Worsening perception of the overall neighborhood environment was associated with increased ST during morning hours (β 0.47; SE 0.19; *p* = 0.01). Additionally, worsening perception of the neighborhood social environment (Factor 4) was associated with increased ST during working hours (β 1.75; SE 0.59; *p* = 0.003). Data for discrete periods of the day are shown in Supplemental Tables 3–5.

Objectively measured non-sedentary time was similarly analyzed. Regression results for total and factor specific NPS for total waking hours and during discrete periods of the day. Results of these analyses were non-significant and are presented in Supplemental Tables 6–9.

## Discussion

Based on cross-sectional data from the bi-racial, socioeconomically diverse HANDLS study, neighborhood environment appears to have a potent association with individuals' PA and ST. The literature on neighborhood environment describes differential effects based on gender (42, 43) and race (44). We considered the potential effect modification of race, sex, and socioeconomic status, but found no significant interactions in our models. This is consistent with Bell and colleagues' findings that gender interactions were only significant for objectively measured neighborhood deprivation and not perceived neighborhood deprivation when assessing the association with BMI and waist circumference (43). In our study, ST was pervasive and PA was low, which demonstrates higher risk of future cardiometabolic disease in this population (3). Although the literature has shown that neighborhoods broadly are associated with health behaviors (45), in this study, we sought to identify specific elements of the neighborhood environment that may be dictating individual PA and ST in a bi-racial, socioeconomically diverse population.

This study is unique in describing precise components of perceived crime, rather than relying on a single metric of general crime perception. Understanding the context in which participants decide to be physically active or sedentary by specifically describing individual types and categories of crime has not been previously done in relation to neighborhood environment, physical activity, and sedentary time and may help focus crime-related interventions. Prior work using agent-based model simulations have shown that radial distance from a crime may be influential in the decision-making process to engage in PA (46). Our findings show that neither higher concern regarding specific types of crime nor higher perceived violent crime at the neighborhood level were associated with PA. This was contrary to our expectations, but has been demonstrated previously in the literature. For example, Oh and colleagues found that neither perceived nor objectively measured crime, nor perceived crime-related safety, were associated with adherence to a walking-based PA intervention among African American women (47). The women with both high and low crime environment scores engaged equally in PA. Additionally, cross-sectional associations between neighborhood environment and PA among low-income African American adults in Pittsburgh demonstrated no significant association between objective neighborhood-level crime at moderate-to-vigorous PA (48). It is possible that participants in our study and others decide to engage in PA in areas away from the home and thereby insulate themselves from the perceived crime (49). Due to the survey-based measure of PA used in this study, we were unable to determine the precise location of the PA. Therefore, we recommend use of objective measures of PA to compliment self-report in future studies.

Another unique aspect of this study is the use of objectively measured ST through accelerometry. Few studies have used accelerometers to capture ST and examine the association with neighborhood factors. Use of an accelerometer allowed us to describe ST patterns throughout the day and over several days for each participant. We found that worse perception of the neighborhood social environment was associated with increased ST overall and between 9 a.m. and 5 p.m., specifically. This may suggest that those who perceive a lack of neighborhood social cohesion may experience limited opportunities to engage positively with neighbors or the surrounding social environment and therefore choose to remain sedentary. Further work using objectively measured ST will be necessary to develop a more comprehensive understanding of sedentary behavior among community-dwelling adults and the influence of neighborhood environment.

Contrary to prior research, our findings do not demonstrate a relationship between perceived neighborhood crime and increased ST (50). Although unexpected, this may point to an underlying relationship between the social environment and the influence of perceived crime on health behaviors. It has been previously shown that there is an inverse association between neighborhood violence and social cohesion (28), where greater social support is associated with higher perceived neighborhood safety among low-income, urban residents (51). Elements of the social environment, including resident social interactions and neighborhood diversity, have been associated with increased walking behavior and decreased ST among older adults (52). Therefore, these findings may suggest the possibility that a positive social environment could temper the impact of perceived crime on health behaviors such as PA and ST. Further investigation is needed to understand what drives individuals' behavior in response to perceived threat of crime and how the social environment may mediate the decision-making process.

The prominent relationship between neighborhood social environment and both PA and ST in our study illuminates a crucial yet physically intangible neighborhood influence on behavior. Improved neighborhood social environment has previously been associated with lower incidence of type 2 diabetes mellitus (53), myocardial infarction (54), and stroke mortality (55). Poor neighborhood social cohesion is hypothesized to act on physical health via transmission of negative health behaviors and lack of social support (45). Therefore, it is possible that improving social cohesion could simultaneously increase PA and decrease ST, but solutions are likely to be community-specific, requiring ongoing dialogue with residents through community engagement, including through community based participatory research (17, 56). Emphasis on opportunities for resident interactions has been shown to facilitate improved neighborhood social cohesion (57) and has been associated with higher probability of meeting PA recommendations (58). Providing communal gathering areas, encouraging mixed land use for walkable destinations, increasing transit stops, facilitating group activities, enhancing multi-generational engagement, and reducing perception of crime would likely improve the social environment and thereby reduce ST (57, 59). Determining which community solutions will succeed may be best accomplished through community engagement (60). The need for community engagement in medical research is well-described by Holzer and colleagues, who demonstrate the potential for enhanced trust and participation (61). Fostering the participant-researcher relationship and including participants as co-researchers offers possibility of more direct tailoring of interventions to community needs based on participant-identified community-specific challenges (62). This may both improve the quality of the research and increase the likelihood of implementation of findings following its conclusion.

This study has several limitations. Foremost, it is lacking in objective crime and physical activity data. While objective measures of neighborhood have been shown to be poorly correlated with an individual's perception (47, 63), having both objective and subjective measures for analysis would enhance our understanding of what is driving the relationships. This study also lacks subjective measures of ST, which would potentially elucidate perceptions of “available” time for PA and thus possibly a critical psychological component of an individuals' decision to engage in PA. Additionally, due to the nature of factor analysis, it is not possible to determine whether there were key items and which items within a factor—for example, what component of the social environment—were responsible for the relationships observed. Knowledge of specific characteristics to be modified will be essential to designing successful, targeted interventions in the future. It must be noted that in this analysis, multiple comparisons were computed without adjusting the *p*-values, therefore the findings should be interpreted with caution. Additionally, the neighborhood violence questions were developed by a HANDLS collaborator and have not been validated as a scale. The questionnaire did not include collision fatalities as an aspect of neighborhood safety, which have been considered as a measure of safety in other studies (64). Finally, due to the cross-sectional nature of the analysis, we are unable to look at health outcomes in this population over time as a result of their exposure to these environmental influences on PA and ST.

## Conclusion and Implications for Health Promotion

Neighborhood socioeconomic status (35) and neighborhood perception (7, 9, 65) are known to be strong determinants of health, specifically chronic disease outcomes (53, 66). This study shows that neighborhood social environment may be influencing residents' behavior and decisions, including the probability of engaging in PA or remaining sedentary for adults living in Baltimore, Maryland. This study is novel in individually highlighting specific elements of the social environment, neighborhood-level crime, and physical neighborhood characteristics which may be key mediators between neighborhoods and health. Furthermore, this study specifically identifies risk factors for increased ST, which are understudied in the literature. Our work, in conjunction with existing literature, is most relevant to designing physical activity and obesity interventions in and around Baltimore, MD (46, 60, 67, 68).

Interventions that focus on improving the neighborhood social environment may enhance residents' perception of PA resources and increase their likelihood of engaging in PA while decreasing the likelihood of ST. Therefore, planning public health interventions to promote PA should not be conducted in isolation, rather, consideration of the neighborhood environment is critical. Addressing the neighborhood environment may be best accomplished through community engagement that results in discussion of specific social environmental barriers. These conversations may take place through the formation of a community advisory board by the research team or through participant focus groups. With respect to conducting research on PA and ST in the community setting, we recommend the use of accelerometers to accurately capture participants' activity throughout the day. Additionally, we recommend the consideration of focused measures of crime to adequately characterize the implications of adverse social behaviors on residents' decision making with respect to PA and ST.

## Data Availability

The datasets for this manuscript are not publicly available due to confidentiality reasons. Requests to access the datasets should be directed to the corresponding author.

## Ethics Statement

The studies involving human participants were reviewed and approved by National Institute of Environmental Health Sciences institutional review board. The patients/participants provided their written informed consent to participate in this study.

## Author Contributions

TP-W conceived of the study, participated in its design, data analysis, and writing the manuscript. SC participated in the study design, data analysis, and writing the manuscript. ES and TH participated in data analysis and drafting the manuscript. CA participated in developing the study design and drafting the manuscript. NM, AZ, and ME oversaw primary data collection for the HANDLS cohort, provided feedback on the study analyses, reviewed analyzed data, and assisted in writing the manuscript.

### Conflict of Interest Statement

The authors declare that the research was conducted in the absence of any commercial or financial relationships that could be construed as a potential conflict of interest.

## Abbreviations

PA: physical activity
ST: sedentary time
CVD: cardiovascular disease
NPS: neighborhood perception score
HANDLS: Healthy Aging in Neighborhoods of Diversity across the Lifespan.
