# Supplementary material for: Cross-Sectional Associations of Neighborhood Perception, Physical Activity, and Sedentary Time in Community-Dwelling, Socioeconomically Diverse Adults
Source: Front Public Health. 2019 Sep 13;7:256. doi: 10.3389/fpubh.2019.00256 (PMC6753201; doi:10.3389/fpubh.2019.00256)
Supplement: Supplementary file 1 [file Table_1.docx]

| Supplemental Table 1. Adjusted linear regression results of total and factor-specific NPS on total self-reported physical activity (n=1359). | | | | | | | | | | |
| --- | --- | --- | --- | --- | --- | --- | --- | --- | --- | --- |
|  | **Model 1** | | **Model 2** | | **Model 3** | | **Model 4** | | **Model 5** | |
|  | β | SE | β | SE | β | SE | β | SE | β | SE |
| Total NPS | **-0.13** | 0.03 | - | - | - | - | - | - | - | - |
| Factor 1 Score | - | - | -0.03 | 0.04 | - | - | - | - | - | - |
| Factor 2 Score | - | - | - | - | **-0.11** | 0.05 | - | - | - | - |
| Factor 3 Score | - | - | - | - | - | - | -0.10 | 0.10 | - | - |
| Factor 4 Score | - | - | - | - | - | - | - | - | **-0.46** | 0.07 |
| Age | **-0.49** | 0.05 | **-0.46** | 0.05 | **-0.47** | 0.05 | **-0.46** | 0.05 | **0.48** | 0.05 |
| Sex | **3.06** | 0.85 | **2.80** | 0.85 | **2.88** | 0.85 | **2.79** | 0.85 | **2.87** | 0.84 |
| BMI | **-0.28** | 0.05 | **-0.28** | 0.05 | **-0.28** | 0.05 | **-0.28** | 0.05 | **-0.26** | 0.05 |
| Race | -0.36 | 0.90 | 0.52 | 0.88 | 0.04 | 0.91 | 0.57 | 0.88 | -0.26 | 0.88 |
| Poverty Status | **-2.84** | 0.87 | **-3.02** | 0.88 | **-2.92** | 0.88 | **-2.99** | 0.88 | -2.64 | 0.84 |
| Education | **5.30** | 0.92 | **5.23** | 0.93 | **5.11** | 0.93 | **5.19** | 0.93 | 4.88 | 0.92 |
| Residence | 1.93 | 1.27 | 1.95 | 1.28 | 2.00 | 1.28 | 1.89 | 1.28 | 1.53 | 1.27 |
| NEI | 0.13 | 0.10 | **0.23** | 0.10 | 0.11 | 0.11 | **0.23** | 0.10 | 0.06 | 0.10 |
| Intercept | **70.69** | 4.45 | **58.95** | 3.67 | **61.63** | 3.82 | **59.54** | 3.74 | **67.48** | 3.73 |
| Bold indicates p<0.05  Model 1: Exposure variable is Total NPS  Model 2: Exposure variable is Factor 1 (Concern about specific types of crime)  Model 3: Exposure variable is Factor 2 (Physical environment)  Model 4: Exposure variable is Factor 3 (Location of violent crime)  Model 5: Exposure variable is Factor 4 (Social environment) | | | | | | | | | | |

| Supplemental Table 2. Adjusted linear regression results of total and factor-specific NPS on self-reported leisure time physical activity (n=1359). | | | | | | | | | | |
| --- | --- | --- | --- | --- | --- | --- | --- | --- | --- | --- |
|  | **Model 1** | | **Model 2** | | **Model 3** | | **Model 4** | | **Model 5** | |
|  | β | SE | β | SE | β | SE | β | SE | β | SE |
| Total NPS | **-0.06** | 0.02 | - | - | - | - | - | - | - | - |
| Factor 1 Score | - | - | 0.008 | 0.03 | - | - | - | - | - | - |
| Factor 2 Score | - | - | - | - | 0.07 | 0.04 | - | - | - | - |
| Factor 3 Score | - | - | - | - | - | - | 0.00003 | 0.07 | - | - |
| Factor 4 Score | - | - | - | - | - | - | - | - | **-0.32** | 0.05 |
| Age | **-0.13** | 0.03 | **-0.12** | 0.03 | **-0.13** | 0.03 | **-0.12** | 0.03 | **-0.14** | 0.03 |
| Sex | 0.90 | 0.56 | **0.75** | 0.57 | 0.82 | 0.57 | 0.75 | 0.57 | 0.82 | 0.56 |
| BMI | **-0.21** | 0.04 | **-0.21** | 0.04 | **-0.22** | 0.04 | **-0.21** | 0.04 | **-0.20** | 0.04 |
| Race | -0.26 | 0.60 | 0.19 | 0.58 | -0.11 | 0.60 | 0.18 | 0.58 | -0.37 | 0.58 |
| Poverty Status | -0.99 | 0.58 | -1.07 | 0.58 | 1.02 | 0.58 | -1.07 | 0.58 | -0.82 | 0.58 |
| Education | **2.87** | 0.61 | 2.79 | 0.62 | **2.77** | 0.61 | 2.81 | 0.61 | 2.60 | 0.61 |
| Residence | 1.17 | 0.85 | 1.16 | 0.85 | 1.21 | 0.85 | 1.17 | 0.85 | 0.89 | 0.84 |
| NEI | 0.07 | 0.07 | 0.11 | 0.07 | 0.06 | 0.07 | 0.11 | 0.07 | 0.01 | 0.07 |
| Intercept | **32.25** | 2.96 | **25.78** | 2.43 | **28.08** | 2.53 | **26.04** | 2.48 | **32.45** | 2.47 |
| Bold indicates p<0.05  Model 1: Exposure variable is Total NPS  Model 2: Exposure variable is Factor 1 (Concern about specific types of crime)  Model 3: Exposure variable is Factor 2 (Physical environment)  Model 4: Exposure variable is Factor 3 (Location of violent crime)  Model 5: Exposure variable is Factor 4 (Social environment) | | | | | | | | | | |

| Supplemental Table 3. Adjusted linear regression results of total and factor-specific NPS on accelerometer-measured sedentary time for morning hours (n=404). | | | | | | | | | | |
| --- | --- | --- | --- | --- | --- | --- | --- | --- | --- | --- |
|  | **Model 1** | | **Model 2** | | **Model 3** | | **Model 4** | | **Model 5** | |
|  | β | SE | β | SE | β | SE | β | SE | β | SE |
| Total NPS | **0.47** | 0.19 | - | - | - | - | - | - | - | - |
| Factor 1 Score | - | - | 0.15 | 0.25 | - | - | - | - | - | - |
| Factor 2 Score | - | - | - | - | 0.50 | 0.33 | - | - | - | - |
| Factor 3 Score | - | - | - | - | - | - | 0.86 | 0.67 | - | - |
| Factor 4 Score | - | - | - | - | - | - | - | - | 0.82 | 0.46 |
| Age | 0.37 | 0.28 | 0.31 | 0.29 | 0.36 | 0.29 | 0.31 | 0.29 | 0.39 | 0.29 |
| Sex | -2.58 | 5.32 | -0.89 | 5.32 | -1.21 | 5.30 | -0.87 | 5.30 | -1.51 | 5.31 |
| BMI | 0.46 | 0.34 | 0.52 | 0.34 | 0.57 | 0.34 | 0.53 | 0.34 | 0.51 | 0.34 |
| Race | -7.87 | 5.58 | -8.90 | 5.68 | -6.35 | 5.76 | -9.51 | 5.67 | -7.11 | 5.65 |
| Poverty Status | 5.47 | 5.33 | 6.32 | 5.36 | 5.78 | 5.35 | 5.99 | 5.35 | 5.64 | 5.35 |
| Education | 3.32 | 5.42 | 3.72 | 5.48 | 4.34 | 5.44 | 4.00 | 5.45 | 4.11 | 5.44 |
| Residence | -6.20 | 8.06 | -6.78 | 8.13 | -7.70 | 8.09 | -6.47 | 8.11 | -6.98 | 8.08 |
| NEI | -0.10 | 0.62 | -0.29 | 0.64 | 0.13 | 0.66 | -0.32 | 0.63 | 0.005 | 0.63 |
| Intercept | **89.71** | 26.82 | **127.89** | 21.82 | **114.89** | 23.52 | **121.46** | 22.39 | **113.74** | 21.16 |
| Bold indicates p<0.05  Model 1: Exposure variable is Total NPS  Model 2: Exposure variable is Factor 1 (Concern about specific types of crime)  Model 3: Exposure variable is Factor 2 (Physical environment)  Model 4: Exposure variable is Factor 3 (Location of violent crime)  Model 5: Exposure variable is Factor 4 (Social environment) | | | | | | | | | | |

| Supplemental Table 4. Adjusted linear regression results of total and factor-specific NPS on accelerometer-measured sedentary time for working hours (n=404). | | | | | | | | | | |
| --- | --- | --- | --- | --- | --- | --- | --- | --- | --- | --- |
|  | **Model 1** | | **Model 2** | | **Model 3** | | **Model 4** | | **Model 5** | |
|  | β | SE | β | SE | β | SE | β | SE | β | SE |
| Total NPS | 0.47 | 0.25 | - | - | - | - | - | - | - | - |
| Factor 1 Score | - | - | -0.10 | 0.33 | - | - | - | - | - | - |
| Factor 2 Score | - | - | - | - | 0.54 | 0.43 | - | - | - | - |
| Factor 3 Score | - | - | - | - | - | - | 0.53 | 0.88 | - | - |
| Factor 4 Score | - | - | - | - | - | - | - | - | **1.75** | 0.59 |
| Age | **0.82** | 0.37 | **0.78** | 0.37 | **0.82** | 0.37 | **0.77** | 0.37 | **0.92** | 0.37 |
| Sex | **17.11** | 6.97 | **19.28** | 6.95 | **18.49** | 6.93 | **18.98** | 6.93 | **17.16** | 6.88 |
| BMI | **1.56** | 0.44 | **1.67** | 0.44 | **1.68** | 0.44 | **1.64** | 0.44 | **1.57** | 0.44 |
| Race | 2.43 | 7.31 | 2.21 | 7.42 | 4.13 | 7.54 | 1.20 | 7.42 | 4.75 | 7.32 |
| Poverty Status | 4.46 | 6.98 | 5.30 | 7.00 | 4.81 | 7.00 | 5.14 | 7.00 | 3.94 | 6.94 |
| Education | -2.39 | 7.10 | -1.54 | 7.15 | -1.43 | 7.11 | -1.75 | 7.12 | -1.57 | 7.04 |
| Residence | -10.25 | 10.57 | -11.40 | 10.63 | -11.79 | 10.59 | -10.80 | 10.62 | -10.81 | 10.49 |
| NEI | -0.37 | 0.81 | -0.42 | 0.83 | -0.12 | 0.85 | -0.54 | 0.82 | -0.05 | 0.81 |
| Intercept | **103.93** | 35.09 | **147.94** | 28.43 | **128.09** | 30.76 | **139.71** | 29.23 | **108.11** | 30.01 |
| Bold indicates p<0.05  Model 1: Exposure variable is Total NPS  Model 2: Exposure variable is Factor 1 (Concern about specific types of crime)  Model 3: Exposure variable is Factor 2 (Physical environment)  Model 4: Exposure variable is Factor 3 (Location of violent crime)  Model 5: Exposure variable is Factor 4 (Social environment) | | | | | | | | | | |

| Supplemental Table 5. Adjusted linear regression results of total and factor-specific NPS on accelerometer-measured sedentary time for evening hours (n=404). | | | | | | | | | | |
| --- | --- | --- | --- | --- | --- | --- | --- | --- | --- | --- |
|  | **Model 1** | | **Model 2** | | **Model 3** | | **Model 4** | | **Model 5** | |
|  | β | SE | β | SE | β | SE | β | SE | β | SE |
| Total NPS | 0.17 | 0.19 | - | - | - | - | - | - | - | - |
| Factor 1 Score | - | - | 0.10 | 0.25 | - | - | - | - | - | - |
| Factor 2 Score | - | - | - | - | -0.24 | 0.32 | - | - | - | - |
| Factor 3 Score | - | - | - | - | - | - | 1.11 | 0.66 | - | - |
| Factor 4 Score | - | - | - | - | - | - | - | - | 0.59 | 0.45 |
| Age | **1.53** | 0.28 | **1.51** | 0.28 | **1.50** | 0.28 | **1.51** | 0.28 | **1.56** | 0.28 |
| Sex | **16.27** | 5.29 | **16.82** | 5.25 | **17.27** | 5.24 | **16.69** | 5.22 | **16.32** | 5.25 |
| BMI | **1.02** | 0.33 | **1.03** | 0.34 | **1.04** | 0.33 | **1.04** | 0.33 | **1.03** | 0.33 |
| Race | -6.14 | 5.54 | -6.67 | 5.60 | -7.36 | 5.70 | -7.77 | 5.59 | -5.37 | 5.58 |
| Poverty Status | 7.04 | 5.39 | 7.37 | 5.29 | 7.58 | 5.29 | 6.96 | 5.27 | 6.88 | 5.29 |
| Education | 2.96 | 5.38 | 2.99 | 5.40 | 3.05 | 5.38 | 3.17 | 5.36 | 3.25 | 5.37 |
| Residence | -7.62 | 8.01 | -7.73 | 8.03 | -7.67 | 8.01 | -7.14 | 8.00 | -7.82 | 8.00 |
| NEI | -0.002 | 0.61 | -0.09 | 0.63 | -0.19 | 0.65 | -0.18 | 0.62 | 0.11 | 0.62 |
| Intercept | **69.98** | 26.61 | **82.49** | 21.48 | **92.69** | 23.27 | **72.04** | 22.02 | **72.08** | 22.88 |
| Bold indicates p<0.05  Model 1: Exposure variable is Total NPS  Model 2: Exposure variable is Factor 1 (Concern about specific types of crime)  Model 3: Exposure variable is Factor 2 (Physical environment)  Model 4: Exposure variable is Factor 3 (Location of violent crime)  Model 5: Exposure variable is Factor 4 (Social environment) | | | | | | | | | | |

| Supplemental Table 6. Adjusted linear regression results of total and factor-specific NPS on accelerometer-measured non-sedentary time for total waking hours (n=404). | | | | | | | | | | |
| --- | --- | --- | --- | --- | --- | --- | --- | --- | --- | --- |
|  | **Model 1** | | **Model 2** | | **Model 3** | | **Model 4** | | **Model 5** | |
|  | β | SE | β | SE | β | SE | β | SE | β | SE |
| Total NPS | -0.79 | 0.45 | - | - | - | - | - | - | - | - |
| Factor 1 Score | - | - | -0.20 | 0.59 | - | - | - | - | - | - |
| Factor 2 Score | - | - | - | - | -0.68 | 0.78 | - | - | - | - |
| Factor 3 Score | - | - | - | - | - | - | -1.68 | 1.60 | - | - |
| Factor 4 Score | - | - | - | - | - | - | - | - | -1.71 | 1.09 |
| Age | **-1.66** | 0.68 | **-1.57** | 0.68 | **-1.63** | 0.68 | **-1.56** | 0.68 | **-1.72** | 0.68 |
| Sex | **-26.19** | 12.70 | **-29.19** | 12.64 | **-28.73** | 12.62 | **-29.09** | 12.59 | **-27.61** | 12.62 |
| BMI | **-2.85** | 0.80 | **-2.96** | 0.81 | **-3.03** | 0.80 | **-2.97** | 0.80 | **-2.92** | 0.80 |
| Race | -3.39 | 13.31 | -1.79 | 13.50 | -5.32 | 13.73 | -0.32 | 13.49 | -5.29 | 13.43 |
| Poverty Status | 14.88 | 12.7 | -16.38 | 12.73 | -15.68 | 12.74 | -15.73 | 12.73 | -14.96 | 12.72 |
| Education | 5.01 | 12.92 | 4.34 | 13.01 | 3.54 | 12.95 | 3.96 | 12.94 | 3.76 | 12.92 |
| Residence | 19.26 | 19.24 | 20.38 | 19.33 | 21.59 | 19.29 | 19.59 | 19.30 | 20.45 | 19.23 |
| NEI | 0.62 | 1.47 | 0.90 | 1.51 | 0.35 | 1.55 | 1.01 | 1.49 | 0.38 | 1.49 |
| Intercept | **662.77** | 63.89 | **597.85** | 51.73 | **615.54** | 56.03 | **612.42** | 53.14 | **630.09** | 55.04 |
| Bold indicates p<0.05  Model 1: Exposure variable is Total NPS  Model 2: Exposure variable is Factor 1 (Concern about specific types of crime)  Model 3: Exposure variable is Factor 2 (Physical environment)  Model 4: Exposure variable is Factor 3 (Location of violent crime)  Model 5: Exposure variable is Factor 4 (Social environment) | | | | | | | | | | |

| Supplemental Table 7. Adjusted linear regression results of total and factor-specific NPS on accelerometer-measured non-sedentary time for morning hours (n=401). | | | | | | | | | | |
| --- | --- | --- | --- | --- | --- | --- | --- | --- | --- | --- |
|  | **Model 1** | | **Model 2** | | **Model 3** | | **Model 4** | | **Model 5** | |
|  | β | SE | β | SE | β | SE | β | SE | β | SE |
| Total NPS | -0.26 | 0.15 | - | - | - | - | - | - | - | - |
| Factor 1 Score | - | - | 0.10 | 0.25 | - | - | - | - | - | - |
| Factor 2 Score | - | - | - | - | -0.24 | 0.32 | - | - | - | - |
| Factor 3 Score | - | - | - | - | - | - | 1.11 | 0.66 | - | - |
| Factor 4 Score | - | - | - | - | - | - | - | - | 0.59 | 0.45 |
| Age | **-0.21** | 0.22 | **1.51** | 0.28 | **1.50** | 0.28 | **1.51** | 0.28 | **1.56** | 0.28 |
| Sex | **1.63** | 4.20 | **16.82** | 5.25 | **17.27** | 5.24 | **16.69** | 5.22 | **16.32** | 5.25 |
| BMI | **-0.32** | 0.27 | **1.03** | 0.34 | **1.04** | 0.33 | **1.04** | 0.33 | **1.03** | 0.33 |
| Race | 3.94 | 4.41 | -6.67 | 5.60 | -7.36 | 5.70 | -7.77 | 5.59 | -5.37 | 5.58 |
| Poverty Status | -1.14 | 4.21 | 7.37 | 5.29 | 7.58 | 5.29 | 6.96 | 5.27 | 6.88 | 5.29 |
| Education | -3.09 | 4.28 | 2.99 | 5.40 | 3.05 | 5.38 | 3.17 | 5.36 | 3.25 | 5.37 |
| Residence | 6.39 | 6.36 | -7.73 | 8.03 | -7.67 | 8.01 | -7.14 | 8.00 | -7.82 | 8.00 |
| NEI | -0.06 | 0.49 | -0.09 | 0.63 | -0.19 | 0.65 | -0.18 | 0.62 | 0.11 | 0.62 |
| Intercept | **96.84** | 21.18 | **82.49** | 21.48 | **92.69** | 23.27 | **72.04** | 22.02 | **72.08** | 22.88 |
| Bold indicates p<0.05  Model 1: Exposure variable is Total NPS  Model 2: Exposure variable is Factor 1 (Concern about specific types of crime)  Model 3: Exposure variable is Factor 2 (Physical environment)  Model 4: Exposure variable is Factor 3 (Location of violent crime)  Model 5: Exposure variable is Factor 4 (Social environment) | | | | | | | | | | |

| Supplemental Table 8. Adjusted linear regression results of total and factor-specific NPS on accelerometer-measured non-sedentary time for working hours (n=404). | | | | | | | | | | |
| --- | --- | --- | --- | --- | --- | --- | --- | --- | --- | --- |
|  | **Model 1** | | **Model 2** | | **Model 3** | | **Model 4** | | **Model 5** | |
|  | β | SE | β | SE | β | SE | β | SE | β | SE |
| Total NPS | -0.47 | 0.26 | - | - | - | - | - | - | - | - |
| Factor 1 Score | - | - | 0.02 | 0.34 | - | - | - | - | - | - |
| Factor 2 Score | - | - | - | - | -0.60 | 0.45 | - | - | - | - |
| Factor 3 Score | - | - | - | - | - | - | -0.62 | 0.93 | - | - |
| Factor 4 Score | - | - | - | - | - | - | - | - | **-1.29** | 0.63 |
| Age | -0.59 | 0.39 | -0.54 | 0.39 | -0.59 | 0.39 | -0.54 | 0.93 | -0.65 | 0.39 |
| Sex | -12.11 | 7.33 | -14.73 | 7.30 | -13.39 | 7.28 | -13.93 | 7.28 | -12.65 | 7.28 |
| BMI | **-1.64** | 0.46 | **-1.72** | 0.47 | **-1.76** | 0.46 | **-1.72** | 7.28 | **-1.67** | 0.46 |
| Race | -4.45 | 7.69 | -3.98 | 7.80 | -6.41 | 7.92 | -3.11 | 7.80 | -6.04 | 7.74 |
| Poverty Status | -4.90 | 7.34 | -5.75 | 7.36 | -5.19 | 7.35 | -5.54 | 7.36 | -4.73 | 7.33 |
| Education | 6.57 | 7.46 | 5.89 | 7.52 | 5.59 | 7.47 | 5.94 | 7.48 | 5.80 | 7.45 |
| Residence | 9.81 | 11.11 | 10.77 | 11.17 | 11.40 | 11.13 | 10.28 | 11.16 | 10.45 | 11.09 |
| NEI | 0.45 | 0.85 | 0.54 | 0.87 | 0.16 | 0.90 | 0.63 | 0.86 | 0.24 | 0.86 |
| Intercept | **345.60** | 36.91 | **303.81** | 29.90 | **323.84** | 32.33 | **311.33** | 30.73 | **332.07** | 31.73 |
| Bold indicates p<0.05  Model 1: Exposure variable is Total NPS  Model 2: Exposure variable is Factor 1 (Concern about specific types of crime)  Model 3: Exposure variable is Factor 2 (Physical environment)  Model 4: Exposure variable is Factor 3 (Location of violent crime)  Model 5: Exposure variable is Factor 4 (Social environment) | | | | | | | | | | |

| Supplemental Table 9. Adjusted linear regression results of total and factor-specific NPS on accelerometer-measured non-sedentary time for evening hours (n=404). | | | | | | | | | | |
| --- | --- | --- | --- | --- | --- | --- | --- | --- | --- | --- |
|  | **Model 1** | | **Model 2** | | **Model 3** | | **Model 4** | | **Model 5** | |
|  | β | SE | β | SE | β | SE | β | SE | β | SE |
| Total NPS | -0.11 | 0.18 | - | - | - | - | - | - | - | - |
| Factor 1 Score | - | - | -0.17 | 0.23 | - | - | - | - | - | - |
| Factor 2 Score | - | - | - | - | 0.31 | 0.30 | - | - | - | - |
| Factor 3 Score | - | - | - | - | - | - | -0.66 | 0.62 | - | - |
| Factor 4 Score | - | - | - | - | - | - | - | - | -0.33 | 0.42 |
| Age | **-0.91** | 0.26 | **-0.89** | 0.26 | **-0.88** | 0.26 | **-0.90** | 0.26 | **-0.93** | 0.27 |
| Sex | **-15.79** | 4.96 | **-15.96** | 4.92 | **-16.60** | 4.91 | **-16.07** | 4.90 | **-15.87** | 4.93 |
| BMI | **-0.79** | 0.31 | **-0.78** | 0.31 | **-0.80** | 0.31 | **-0.80** | 0.31 | **-0.80** | 0.31 |
| Race | -1.39 | 5.20 | -0.70 | 5.25 | 0.01 | 5.34 | -0.42 | 0.31 | -1.81 | 5.24 |
| Poverty Status | -8.45 | 4.97 | -8.70 | 4.97 | -8.95 | 4.96 | -8.42 | 5.25 | -8.39 | 4.97 |
| Education | 0.39 | 5.05 | 0.59 | 5.06 | 0.42 | 5.04 | 0.26 | 5.04 | 0.21 | 5.04 |
| Residence | 4.12 | 7.52 | 3.96 | 7.53 | 3.98 | 7.51 | 3.85 | 5.72 | 4.25 | 7.51 |
| NEI | 0.12 | 0.57 | 0.23 | 0.59 | 0.34 | 0.60 | 0.23 | 0.58 | 0.06 | 0.58 |
| Intercept | **225.46** | 25.00 | **219.84** | 20.14 | **206.06** | 21.81 | **223.55** | 20.70 | **223.20** | 21.49 |
| Bold indicates p<0.05  Model 1: Exposure variable is Total NPS  Model 2: Exposure variable is Factor 1 (Concern about specific types of crime)  Model 3: Exposure variable is Factor 2 (Physical environment)  Model 4: Exposure variable is Factor 3 (Location of violent crime)  Model 5: Exposure variable is Factor 4 (Social environment) | | | | | | | | | | |
